# Prospects and Challenges of Induced Pluripotent Stem Cells in Equine Health

**DOI:** 10.3389/fvets.2015.00059

**Published:** 2015-11-19

**Authors:** F. Xavier Donadeu, Cristina L. Esteves

**Affiliations:** ^1^The Roslin Institute and Royal (Dick) School of Veterinary Studies, University of Edinburgh, Midlothian, UK

**Keywords:** horses, stem cells, induced pluripotent stem cells, regenerative medicine, cell differentiation

## Abstract

Pluripotent stem cells (PSCs) hold, through the capacity to differentiate into virtually all body cell types, unprecedented promise for human and animal medicine. PSCs are naturally found in the early embryo, and in rodents and humans they can be robustly harvested and grown in culture in the form of embryonic stem cells (ESCs); however, the availability of ESCs from horses is limited. ES-like cells named induced pluripotent stem cells (iPSCs) can be derived *in vitro* by transcription factor-mediated reprogramming of adult cells. As such, iPSCs can be generated in a patient-specific manner providing unmatched potential for tissue transplantation and *in vitro* disease modeling. In humans, clinical trials using iPSC-derived cells are already taking place and the use of *in vitro* iPSC models has identified novel mechanisms of disease and therapeutic targets. Although to a more limited extent, iPSCs have also been generated from horses, a species in which, after humans, these cells are likely to hold the greatest potential in regenerative medicine. Before a clinical use can be envisioned, however, significant challenges will need to be addressed in relation to the robust derivation, long-term culture, differentiation, and clinical safety of equine iPSCs. Toward this objective, recent studies have reported significant improvement in culture conditions and the successful derivation for the first time of functional cell types from equine iPSCs. Given the wide range of exciting applications they could have, it is hoped future research will make the biomedical promise of iPSCs a reality not only for humans but also horses.

Stem cells are defined based on their capacity for self-renewal and the ability to differentiate into specialized cell types (potency). In contrast to multipotent stem cells (including mesenchymal stem cells or MSCs), pluripotent stem cells (PSCs) are intrinsically able to self-renew indefinitely and to give rise to virtually all cell types in the body, features that provide distinct advantages in relation to regenerative medicine applications and for which PSCs have been the subject of intense research over the past 30 years. Although pluripotent cells are found naturally only in the early mammalian embryo, pluripotency can be captured *in vitro* in the form of embryonic stem cells (ESCs) generated from cultures of the inner cell mass, the forerunner of the embryo proper in the very early conceptus (1, 2). ESC lines that maintain their pluripotency *in vivo*, i.e., are able to give raise to differentiated teratomas when injected into immunodeficient mice, have been robustly derived from rodents and humans but not, to this date, from domestic species, including the horse. This is partly attributed to the relative lack of knowledge of early embryo development in domestic species, which precludes the use of optimal conditions to stably maintain embryonic cells in an undifferentiated state. Nonetheless, cultures of equine embryonic cells that lack pluripotency *in vivo* have been established by several groups (3, 4) and their potential in relation to veterinary regenerative medicine is being investigated (5, 6).

## Generation and Characterization of Equine iPSCs

In 2006, Shinya Yamanaka’s group in Japan showed that cells equivalent to ESCs, named induced pluripotent stem cells (iPSCs), could be generated in culture from murine fibroblasts by simply inducing the expression of four genes, namely the pluripotency-associated transcription factors, Oct4, Sox2, Klf4, and Myc (7). This seminal discovery was followed shortly after by the successful generation of iPSCs from humans (8) and opened the way to the derivation, without the need to use embryos, of patient-specific PSCs that could be used for autologous tissue transplantation, thus, providing a clear advantage over ESCs. For his discoveries, Yamanaka was awarded the 2012 Nobel Prize in Medicine.

The first reports on mouse and human iPSCs in 2006–2007 led to a deluge of studies aiming to identify cell sources and gene expression systems that would allow efficient reprogramming using minimal genetic modification of the resulting iPSCs, a crucial requisite for an eventual clinical application of these cells. Studies soon extended to domestic animal species where iPSC technology was seen as a highly promising alternative to ESCs (9–12). In the horse, the prospect of a new source of stem cells for clinical use led to the first report on equine iPSCs in 2011 (13) followed by several additional publications over the following 3 years (14–17). The cells generated in these studies displayed, at various levels, features of equine embryonic cells and iPSCs from mice and humans (Table 1), including morphology, re-activated expression of molecular markers of pluripotency and the ability, in some studies, to generate differentiated teratomas *in vivo*, which is to-date the most stringent proof of pluripotency in the horse. Equine iPSCs were generated from fetal or adult fibroblasts and, in one of the studies, keratinocytes (Table 1); consistent with reports in humans (18), equine keratinocytes were relatively more amenable to reprogramming than fibroblasts and, in addition, once reprogrammed they had higher developmental plasticity than fibroblast-derived iPSCs as indicated by their ability to produce differentiated tissues *in vivo* as diverse as neurons, cartilage, muscle, lung epithelium, and gastric epithelium (16).

**Table 1:** **Characteristics of reported equine iPSCs**.

	Nagy et al. (13)	Breton et al. (15)	Khodadadi et al. (14)	Sharma et al. (16)	Whitworth et al. (17)
Reprogramming cell source	Dermal fibroblasts, 55-day-old fetus	Dermal fibroblasts, 5-month-old foal and 2-year-old gelding	Fibroblasts, adult horse	Keratinocytes, 5-month-old filly	Dermal Fibroblasts, adult mare
Reprogramming system	piggyBac transposon containing murine *Oct4, Sox2, Klf4* and *Myc*, tetracycline-driven	MMLV vector expressing murine *Oct4, Sox2, Klf4* and *Myc*,	MMLV vector expressing human *OCT4, SOX2*, and *KLF4*	MMLV vector expressing murine *Oct4, Sox2, Klf4* and *Myc*	Lentivirus vector expressing human *OCT4, SOX2, KLF4*, and *MYC*
Culture conditions	DMEM high glucose, FBS, LIF, bFGF, CHIR99021, PD0325901, A83-01, Thiazovivin, SB431542	Knock-out (KO) DMEM, KO serum replacement, LIF, bFGF	α-MEM, FBS, ITS, LIF, bFGF, EGF	Equine fetal fibroblast-conditioned media (KO DMEM, KO serum replacement), LIF	KO DMEM, FCS, LIF
	MEF:EEF feeders (1:1)	SNL feeders	MEF feeders	Feeder-free	MEF feeders
Pluripotency markers expressed ^a^	ALP, NANOG, *OCT4, KLF4*, SSEA1, SSEA4, TRA1-60, TRA1-81	ALP, OCT4, SOX2, NANOG, REX1, LIN28, *DNMT3B*, SSEA1, SSEA4, TRA1-60	ALP, OCT4, *SOX2*, NANOG, *STAT3*, SSEA1, SSEA4	ALP, OCT4, SOX2, *NANOG, REX1*, LIN28, *DNMT3B*, SSEA1	ALP, NANOG, *OCT4, TERT*, REX1, SSEA4, TRA1-60, TRA1-81
Differentiation potential					
*In vitro*	Not tested	Ectoderm, mesoderm, endoderm	Ectoderm, mesoderm, endoderm	Ectoderm, mesoderm, endoderm	Ectoderm, mesoderm, endoderm
*In vivo*	Ectoderm, mesoderm, endoderm	Ectoderm, mesoderm, endoderm	Ectoderm, mesoderm, endoderm	Ectoderm, mesoderm, endoderm	Not tested
Clinically relevant lineages generated	Keratinocytes (19)	Not reported	Not reported	Functional motor-neurons	Not reported

*^a^Gene symbols are shown according to current HGNC guidelines (http://www.genenames.org/about/guidelines#criteria)*.

*MMLV, Moloney murine leukemia virus; FBS, fetal bovine serum; LIF, leukemia inhibitory factor; bFGF, basic fibroblast growth factor; MEF, mouse embryonic fibroblasts; EEF, equine embryonic fibroblasts; ITS, insulin–transferrin–selenium; EGF, epidermal growth factor*.

Reprogramming of equine cells was achieved in most studies by using viral expression vectors that mediate the integration of the reprogramming gene sequences (Oct4, Sox2, Klf4, and Myc) into the cell genome, therefore, making the reprogrammed cells not apt for clinical use. Only one study (13) used a non-integrating expression vector (piggyBac transposon) to reprogram equine cells, although this was done at the cost of reduced robustness of the resulting iPSCs, as switching off the expression of the reprogramming DNA sequences in these cells led to loss of pluripotency and rapid differentiation, indicating that re-activated expression of endogenous pluripotency genes during reprogramming was not sufficient to sustain the pluripotent state. Consistent with this observation, all other iPSC lines reported to date show clear but variable expression of the reprogramming genes, similar to observations from other domestic species (9, 11, 12). Given that silencing of the exogenous reprogramming genes is in general considered a hallmark of faithful reprogramming (20), the above findings bring into question whether equine iPSC lines reported so far are fully reprogrammed or they represent instead a partially reprogrammed cell type; potential implications of this, if any, in relation to a possible clinical application of these cells need to be established.

## Potential of iPSCs in Equine Biomedicine

Induced pluripotent stem cells offer truly unprecedented potential both as a source of therapeutic cells and as a tool for *in vitro* disease modeling and drug discovery. The tissue regeneration potential of iPSCs has been demonstrated using different animal models of disease, including spinal cord injury (21), Parkinson (22), and retinal degeneration (23). Notably, the first human clinical trials using autologous iPSCs are already underway in Japan to treat age-related macular degeneration, and significant work is being carried out toward other eventual clinical applications. A particularly exciting possibility in this regard involves the use of iPSCs in combination with novel gene editing technologies as a new strategy for gene therapy; although a long-term clinical prospect, success using this combined approach has been reported in experimental rodent models, including sickle cell anemia (24) and limb-girdle muscular dystrophy (25).

Very early steps have been given to test the clinical efficacy and safety of PSCs in the horse. Guest et al. (5) injected equine ESC-like cells into damaged tendon of live horses and showed superior cell survival and migration to damaged tissue compared to transplanted bone marrow-derived MSCs. A more recent study showed intradermal injection of equine allogeneic iPSCs to be relatively well-tolerated and without noticeable long-term effects (26). These reports are encouraging and further studies will need to test the potential of iPSCs in relation to specific disorders in the horse. Clinically, equine iPSCs may be most useful in the first instance as an alternative to current MSC-based musculoskeletal therapies. In this regard, significant benefits can be gained from progress made with humans for which different protocols are already available to derive MSC-like cells from iPSCs (27–29), moreover, the potential of such cells for tissue regeneration has been clearly demonstrated using mouse models of limb ischemia (27, 29). Although, in principle, iPSCs could be derived from any equine patient, the time required for the generation and pre-clinically testing of such cells will prevent their therapeutic use early during the disease course, making autologous application a suitable option only for some non-acute disorders. Indeed, equine iPSCs will have greatest clinical potential as an off-the-shelf source of therapeutic, *in vitro*-produced mesenchymal precursors, a possibility that holds particular attractive at a moment when allogeneic cell therapies are being increasingly considered in the horse. Off-the-shelf iPSC-derived cells could have applications not only in musculoskeletal repair but also for other common conditions, including external wounds, ischemic/inflammatory processes such as laminitis or gastro-intestinal disease, autoimmune disorders, and even neuro-regeneration (16, 19).

Despite their enormous clinical promise, given present concerns about overall clinical safety (discussed below), disease modeling is seen as the application for which iPSCs, in general, will be most useful in the short term. In that regard, the differentiation of human patient-derived iPSCs into functional cells capable of recapitulating specific disease phenotypes has provided a truly unmatched tool for studying diseases *in vitro*. Disorders successfully modeled in this way include neurological, cardiovascular, hematological and metabolic (30); such studies have unraveled novel molecular mechanisms of disease as well as led to identification of promising therapeutic targets. Most notably, elegant studies [reviewed in Ref. (31)] with human iPSC-derived cardiomyocytes and neurons have elucidated novel genetic mechanisms in the pathogenesis of long-QT syndrome and familial dysautonomia, respectively. Moreover, using neurons from patients with familial dysautonomia or Rett syndrome led to the identification of novel therapeutic compounds that are now being used in early-stage clinical trials (31).

A similar potential for *in vitro* modeling of disease as well as normal tissue development is held by equine iPSCs. This includes the possibility, using appropriate iPSC-derived cell types, of studying the effects of different genetic backgrounds or horse breeds on disease resistance and the responses to specific therapies. This information could be extremely valuable for developing new therapies or improving current approaches for equine patients. The limited knowledge, both basic and applied, on developmental/stem cell biology and molecular aspects of disease in the horse will certainly pose significant challenges to such application. These include the need to establish robust differentiation protocols to produce cells phenotypically equivalent to the cell type(s) under study, and to appropriately identify and measure phenotypes that relate to the diseases of interest, which may be species specific. Diseases for which a phenotype is most likely to be obtained *in vitro* and for which iPSCs may be most useful are early-onset, cell-autonomous defects caused by known, high penetrance mutations. Several monogenic diseases with a defined causal mutation have been identified in horses (http://omia.angis.org.au). Some, such as hyperkalemic periodic paralysis and exertional rhabdomyolysis, have a relatively high, breed-specific prevalence and could be readily targeted. In that case, the phenotype of myocytes derived from iPSCs from affected and healthy animals would be compared to understand the cellular mechanisms underlying the disease(s) and establish the responses to potential therapeutic agents; alternatively, myocytes could be derived from healthy iPSCs in which the causal gene mutation would have been previously induced and would be compared to myocytes generated from non-mutated iPSCs (30). Advances in sequencing of the equine genome, together with the development over the past few years of readily available technologies for precise genome editing will significantly facilitate such undertakings toward making real therapeutic progress in relation to those and many other equine diseases.

## Challenges for the Application of iPSCs in Equine Biomedicine

In spite of their enormous potential, several challenges related to clinical safety will first need to be overcome before a therapeutic use of equine iPSCs can be considered. The most important concern at present is the possibility of tumor formation following transplantation of iPSCs into patients. The capacity to generate a wide variety of unwanted tissue types after transplantation, including malignant tumors derives from the inherent development plasticity of all pluripotent cells. In addition, the reprogramming process itself together with the repeated passaging of the reprogrammed cells in culture can lead to significant genetic and epigenetic instability and an increased propensity for tumor formation (32). Another very important consideration is that iPSCs are typically generated by inducing the expression of gene sequences (Oct4, Sox2, Klf4, and c-Myc) which by themselves can be tumorigenic. As already pointed out, reprogramming is most efficiently achieved by the use of viral vectors carrying the reprogramming genes that become permanently integrated into the cell’s genome, resulting in reprogrammed cells that harbor foreign, potentially tumorigenic DNA. In addition, the continued expression of these genes can impinge on the ability of the iPSCs to later differentiate properly into adult cell types (consistent with the notion that silencing of the reprogramming genes is a hallmark of successful reprogramming), thus, reducing their clinical applicability (21). Efforts to circumvent these issues have resulted in the development of several non-integrating expression systems (33, 34), which can now be used routinely to generate human iPSCs with reasonable efficiency. Based on results (13) using a piggyBac transposon system to reprogram equine fibroblasts and on several reports in other domestic species [reviewed in Ref. (35)], it is likely that significant optimization of reprogramming and culture conditions will be necessary to efficiently derive robust equine iPSCs using available non-integrating expression systems. Such efforts will be essential for any eventual clinical application of these cells. In addition, thorough molecular and functional characterization and testing of individual iPSC lines intended for clinical use will be mandatory to completely rule out genetic or phenotypic traits indicative of tumorigenic capacity. Despite all these challenges, studies in other species have already reported the successful transplantation of iPSC-derived cells without any resulting tumorigenicity or immunogenicity (21).

Related to the point above, a significant challenge toward an eventual clinical use of equine iPSCs will be the development of robust protocols for unidirectional and efficient differentiation to specific mature and functional cell types. Significant progress has already been made in human, rodent, and even pig (30, 36), iPSCs from which species can now be differentiated into cells resembling, with variable fidelity, many different body cell types; a remaining limitation in that regard is that iPSC-derived cells often present immature, fetal-like phenotypes that may not be suitable for tissue transplantation, requiring future refinement of cell differentiation protocols. In the case of the horse, an additional limitation is the relatively lack of knowledge of normal cellular and molecular mechanisms of development, the recapitulation of which *in vitro* may be required to robustly produce cell lineages of interest. Despite this, progress has already been made with the successful generation for the first time of functional cells from iPSCs, namely 1) functional motor-neurons (16) and 2) cells that both expressed keratinocyte markers and were able to epithelize wounds *in vitro* (19). These achievements have provide important proof of concept of the biomedical potential of equine iPSCs and should encourage further exploratory studies.

The immunogenicity of transplanted iPSC derivatives will also need to be addressed. Although not tested in the horse, studies in mice and primates (37, 38) have shown that transplantation of autologous iPSC-derived cells induces minimal immune response, in contrast to the substantial activation and infiltration of immune cells leading to reduced survival of transplanted allografts. In that regard, although encouraging, the recent report that intradermal injection of allogeneic equine iPSCs produced only a moderate immune response at the site of injection needs to be interpreted with caution as the long-term fate of the injected cells was not established (26). As a clinical use of iPSCs in the future is more likely to occur in an allogeneic context, specific strategies to induce immune tolerance may need to be developed. The creation of human iPSC haplobanks from individuals homozygous at the major HLA antigens has been proposed as a route to provide immuno-compatible cells for a large proportion of the human population (39); whether such an ambitious approach would be necessary, and feasible, in the horse remains to be seen.

Finally, in addition to safety considerations, the allogeneic use of equine iPSC derivatives would likely come under regulatory scrutiny, including Good Manufacture Practice guidelines for their derivation, storage, and application in patients. While steps already being given in the human field could be followed, the implementation of such measures would require a much better understanding of the nature of equine iPSCs and a significant optimization and validation of the procedures currently used for their generation, culture, and differentiation.

In conclusion, although significant challenges in relation to an eventual safe therapeutic application of iPSCs remain ahead, through their demonstrated clinical and *in vitro* modeling potential, iPSCs provides an opportunity to make unprecedented strides toward understanding and treating a variety of equine diseases. Such perspectives should stimulate work by the equine scientific community toward a better understanding of the nature and biomedical potential of these cells.

## Author Contributions

Both authors have contributed to the writing of this review.

## Conflict of Interest Statement

The authors declare that the research was conducted in the absence of any commercial or financial relationships that could be construed as a potential conflict of interest.
